# Characterization of oral cancer diagnostic delay in the state of Alagoas

**DOI:** 10.1590/S1808-86942010000400002

**Published:** 2015-10-19

**Authors:** Luiz Carlos Oliveira dos Santos, Olívio de Medeiros Batista, Maria Cristina Teixeira Cangussu

**Affiliations:** a Doctoral thesis in stomatology, Integrated Doctoral Program in Dentstry, UFPB/UFBA. Adjunct professor of stomatology, UFAL; b Doctoral thesis in sciences biologiques et santé, Rennes I University (URI), France. Adjunct professor of stomatology, UFPB; c Doctoral thesis in public health, Sao Paulo University. Adjunct professor in the Social and Pediatric Dentstry Department

**Keywords:** diagnostic services, epidemiology, mouth neoplasms

## Abstract

Oral cancer in Brazil still presents high levels of incidence and mortality bearing different traits throughout the national territory. In most of the cases the diagnosis is late; however there is a great possibility for cure when treated early on.

**Aim:**

to assess factors associated with the late diagnosis of oral cancer in the state of Alagoas.

**Material and Methods:**

a prospective cross-sectional study was carried out in 74 patients, all of them diagnosed with oral squamous cell carcinoma in a hospital of Alagoas, between July of 2007 and September of 2008. A semi-structured interview was given, obtaining socio-demographic data, the type of professional help sought, symptom onset, referrals and tumor clinical stage at the moment of diagnosis.

**Results:**

According to the results obtained in this study, the patients usually sought professional medical help, rather than dental help when a lesion in the mouth appeared, being always referred to a specialist by the dentist, in advanced stages of the disease.

**Conclusions:**

This study suggests the need for continued education programs for the population and professionals aiming at the early identification of symptoms of the illness; however needing further studies.

## INTRODUCTION

The purpose of an epidemiological study of mouth cancer has been an ongoing problem because of health and quality of life issues in patients.^1^

Epidemiology allows us to state that the incidence of mouth cancer is high worldwide; it has become a public health issue, for which prevention and early diagnosis are the best approaches to revert this situation.^2^

The mouth can be easily accessed for examination purposes, making is possible for dental surgeons, general practitioners, and even patients themselves (by self-examination) to visualize suspected alterations, particularly in their early stages, for the purpose of an early diagnosis. In most cases, however, the diagnosis is delayed.^3^^,^^4^

An early diagnosis is not necessarily easy, because patients and healthcare professionals underrate the initial lesions, which are generally asymptomatic. This reality suggests that physicians have gaps in their knowledge of pathology, that patients delay seeking medical care, and that access to and the quality of medical care is deficient, which reflect the absence of preventive public health programs and an effective healthcare system.

An increasing number of cases of cancer have been identified in the state of Alagoas, in a context of delayed diagnoses and poor access to treatment; 110 new cases of mouth cancer alone were diagnosed in 2008,^5^ which has raised the number of incurable cancer patients. These patients are generally sent to their homes where they are in pain and suffer other symptoms of this disease until their deaths.^6^

As mentioned above, most of the cases of mouth cancer are diagnosed when the disease is at an advanced stage; it is thus necessary to find the factors that will have led to this situation. In this context, a study was carried out to establish and analyze such factors, thereby helping medical referral units to become better organized for dealing with mouth cancer in this state.

## METHODS

A prospective exploratory quantitative study was carried out in a sample of 74 squamous cell carcinoma patients diagnosed at a state hospital in Alagoas from July 2007 to September 2008 with the purpose of investigating the epidemiological profile of these patients.

The survey was undertaken at the Head & Neck Surgery Unit of the aforementioned hospital, which is a reference center in oncology in Alagoas, receiving patients from 102 municipalities grouped into 13 healthcare micro-regions.

An individual questionnaire with the following variables was applied: age, gender, marital status, race, profession, education level, habits (smoking and drinking alcohol), duration of initial disease symptoms, disease progression time (in months), whether visited a neighboring healthcare unit, the time elapsed until referral to a specialized unit, the healthcare professional that was sought, the medical approach and tumor staging.

Patients with a diagnosis of mouth cancer, seen at the outpatient unit by a hospital physician (head & neck surgeon or oncologist) signed the free informed consent form and were interviewed by the stomatologist (researcher in charge).

For the purpose of this study, a late diagnosis was that made three weeks or more after the onset of symptoms; the public health units recommend that patients with “lesions or ulcers” that do not heal within two weeks should visit a healthcare unit.^7^

Tumors were recorded based on the Classification of Oncological Diseases (ICD-O) and the TNM classification of the International Union Against Cancer or IUAC. Tumors are defined as advanced when in stages T3 or T4 according to the T classification. Tumors were staged as I-II (T1 or T2 and N0) and III-IV (T3, T4 or N>0).^8^

A late diagnosis was counted in months from the date of onset until the diagnosis at a referral healthcare unit and tumor staging at the time of diagnosis.

The data were stored in a Windows Excel 2003 database; a descriptive analysis consisted of central tendency measures and dispersion for continuous variables, and the number and percentage of categorical variables. The chi-square test and Student's t test (at a 95% significance level) were applied to test the differences among groups.

This study had formal approval from all sectors involved, and abided by the current ethical standards as defined by the Resolution 196/96 of the National Health Council; the identity of patients and healthcare professionals remained confidential. The patients were free to participate or not in this study without any effect on their subsequent therapy.

The institutional review board assessed and approved this study (no. 005567/2006-17).

## RESULTS

### Characterizing the Study Population

The sample consisted of the files of 74 patients that sought the healthcare unit in a 14-month period. The mean age was 57.22 years (SD=14.01), ranging from 26 to 85 years. Table 1 shows other social and demographic data.

**Table 1:** Social and demographic features and habits of patients presenting at the Head & Neck Unit outpatient unit of the Holy House of Mercy Hospital (Hospital Santa Casa de Misericórdia) in Maceio, Alagoas, 2008.

Variables	N	%	Test c^2^	P value
Gender				
1. Male	52	70,3	8,89	0, 003
2. Female	22	29,7		
Race				
1. White	28	37,8	3,96	0,047
2. Non-white	46	62,2		
Age				
1. 26 – 59 years	35	47,3	0,49	0,49
2. 60 years or over	39	52,7		
Education				
1. Illiterate	28	37,8	1,70	0,43
2. Literate	14	18,9		
3. Basic education or over	32	40,3		
Work				
Rural worker	29	39,2	0,83	0,37
Housewife	5	6,8		
Biscateiro	5	6,8		
Fisherman	4	5,4		
Other	31	41,8		
Smoking and drinking alcohol				
1. Smokes and drinks	29	39,1	0,87	0,32
2. Only smokes	28	37,8		
3. Only drinks	2	2,7		
4. None	15	20,3		
Family income				
< 1 minimum salary	15	20,3	16,35	0, 000
1 minimum salary	49	66,2		
2 or more minimum salaries	10	13,5		

c^2^ = chi-square statistics

N = number of patients

Of the 74 patient, 70.3% were male 29.7% were female; males were statistically more numerous than females.

The education level showed a balance between illiterate persons (37.8%) and those with incomplete basic education or over (40.3%). Rural workers comprised 39.2% of the sample.

The family income of most subjects ranged from less than a minimum salary (20.3%) to a minimum salary (66.2%), notwithstanding their social and demographic features.

Patients were referred from 31 municipalities in Alagoas, which corresponds to 30.4% of the 102 municipalities of that state; most patients came from the capital Maceio, followed by Arapiraca.

Fifty-two patients (70.3%) had sought a physician before being referred to the hospital in Alagoas, 21 patients (28.4%) had sought a dentist, and one patient (1.3%) had sought a community healthcare agent.

**Figure 1:**
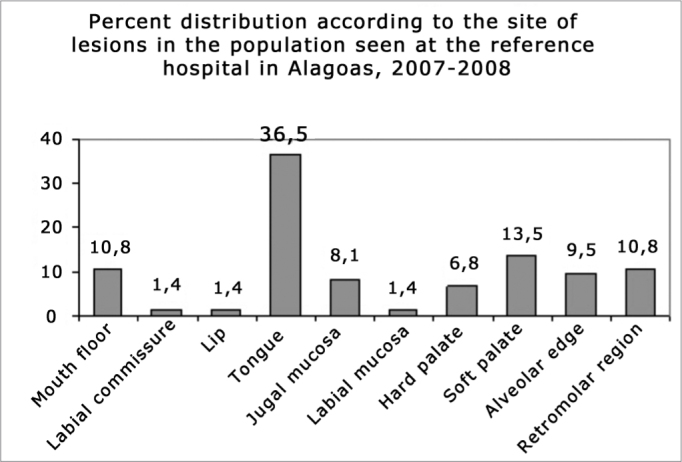
Anatomical site of lesions.

The prevalence of disease in the tongue was statistically higher compared to other sites (Fig. 1).

Most patients (78.4%) had tumors staged above T2N0M0 (stages III and IV), which characterized advanced disease. T1N0M0 and T2N0M0 were defined in 21.6% of patients, corresponding to stages I and II (therefore, early disease). Advanced disease predominated in most patients.

**Figure 2:**
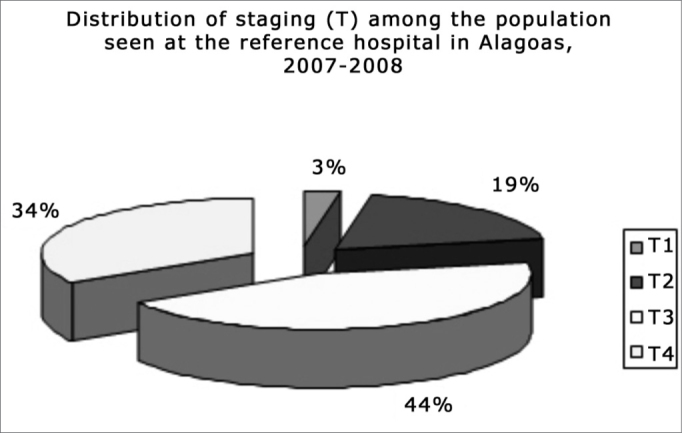
Distribution of staging as detected.

Figure 2 shows the stages of disease. Lesions at advanced stages predominated, which was statistically significant compared to stages T1 and T2 (k^2^= 37.33; p< 0.00)

### Diagnosis and Referral to Healthcare Units

The reported time by patients from the onset of symptoms to the first visit to a specialist at the Head & Neck Unit of the reference hospital in Alagoas was 1-3 months (24 patients – 32.4%), 4-6 months (20 patients – 27.0%), 7-9 months (20 patients – 27.0%), and over 9 months (10 patients – 13.5%).

The time elapsed between the first visit to the referring physician at the place of origin of patients and the visit to the Head & Neck Unit of the reference hospital in Alagoas was less than 2 months (20 patients – 27.0%), 2-6 months (41 patients – 55.4%), and over 6 months (13 patients – 17.6%).

Information about the referral to a reference hospital made by a physician or dentist of patients with early or advanced disease, a dentist referred 6 patients (8.1%) with early disease and 15 patients (20.3%) with advanced disease, totaling 21 patients (28.4%). A physician referred 10 patients (13.5%) with early disease and 42 patients (56.8%) with advanced disease, totaling 52 patients (70.3%); a healthcare community agent referred one patient (1.4%) with advanced disease (T3). Thus, healthcare professionals referred most patients; a significant number of the cases were at an advanced stage of the disease upon referral.

The open question yielded homogeneous thought categories; a method used previously in the state of Paraiba^9^ was applied to select similar responses expressing the same ideas.

Five categories were defined based on the answers of patients about the reason for delaying their visit to healthcare centers; these are shown on Table 2.

**Table 2:** Frequency of responses to the open question: Why did you not seek health care before?

Categories	N	%	k^2^	^p^
- Knew of the lesion but only sought care because of pain	31	41,9	6,24	0,10
- Sought health care but were erro-neously diagnosed and treated	18	24,3		
- Did not know of the lesion and sought care because of pain.	2	2,7		
- Denied the disease and treatment	14	18,9		
- Difficult to reach health care	9	12,2		
Total	74	100,0		

The category with most patients (41.9% of the total) was the group that reported they knew about the lesion but sought healthcare only when it started to bother. There was not statistical difference among the answer percentages (p=0.10).

## DISCUSSION

The most frequent type of mouth cancer in the study sample was the squamous cell carcinoma (100% of cases), which is similar to published results.^10^ Because of its significant incidence, papers on this histopathological type merge with those dealing with mouth cancer in general; our discussion, therefore, will deal with mouth cancer as a whole.^11^

Of the 102 municipalities in Alagoas, only 31 referred mouth cancer patients to the reference hospital in that state. Most originated from the capital city, followed by the city of Arapiraca. This is similar to another survey done in Alagoas,^12^ which underlined the importance of medical services in the capital city and the lack of decentralized middle and high complexity healthcare units in Alagoas. The absence of municipalities in the aforementioned statistics may be explained by geographic distance and poor transportation in many rural towns, besides the lack of an effective referral and counter-referral system between basic healthcare and reference hospitals for the treatment of cancer.

There were 74 patients diagnosed with mouth cancer during the study period, which were seen at the reference hospital. Lesions predominated at ages over 60 years, which was generally similar to published results,^13^, ^14^, ^15^ except for some authors who found a higher predominance of disease in the 50-60 years age group.^16^, ^17^, ^18^, ^19^

The sample consisted mostly of male patients; this male predominance in dentistry for this disease may be seen in several studies.^5^^,^^14^, ^15^, ^16^^,^^18^ This trend in male patients has been observed frequently; in recent decades, however, there has been an increase in the incidence of mouth squamous cell carcinoma in females, because of behavioral changes.^19^

There was a high rate of patients with less than basic education (42 patients), of which 28 patients were illiterate. This number is lower than the data in a study in the state of Paraiba (2006)9 where 75% of interviewees were illiterate. Most patients were rural workers, again similar to the data in other published papers.^16^^,^^19^

A higher incidence was found in non-whites, which is dissimilar to several findings in the literature,^20^ where whites predominated, but similar to other papers.^21^ It has been suggested that involvement by race varies depending on the study method and the region,^22^ which makes comparisons difficult.

Among the sample, 37.8% smoked tobacco cigarettes, 39.1% smoked and consumed alcoholic drinks, and 20.3% neither smoked not drank alcohol. Note that these habits are frequent in this sample. Smoking and drinking alcohol have often been reported as the main risk factors for mouth cancer; both habits operate synergically, thereby increasing the incidence of this disease.^21^, ^22^, ^23^, ^24^

The tongue was the most common site, followed by the soft palate, which is similar to published results in the literature,^15^^,^^18^^,^^19^ but which differs from some papers.^25^^,^^26^

Most patients had clinical advanced tumors (T3 or T4), which is similar to some published studies,^9^^,^^12^ suggesting that patients arrive at healthcare centers with advanced stage disease.

Several factors may affect the quality of care for cancer patients, such as promptness of care and availability of material and professional resources. However, even when these resources are present, their effect on the outcome is weak; patient survival is more strongly related with an early diagnosis of this disease.

According to several authors,^4^^,^^27^^,^^28^^,^^29^ the time elapsed between the perception of symptoms and correct diagnosis and treatment affects the outcome and the quality of life of patients.

Our data revealed that 41.9% of patients knew they had a lesion but only sought healthcare when symptoms arose (Table 1). This was the largest category, suggesting lack of self-care in seeking therapy and poor knowledge about the signs of this lesion, which are usually asymptomatic to begin with. A considerable percentage of in-correct treatments showed that healthcare professionals were unprepared to deal with mouth cancer. Similarly, denial of disease underlines its negative stigma among the population, which generally regards cancer as a death sentence with no reprieve - not the case when the disease is diagnosed early. Difficulty in accessing the unified healthcare system (SUS) for middle and high complexity care was evidenced in 12.2% of cases, showing that the system is not easily available for these workers.

Fifty-two patients were first seen by a physician, and 21 patients were first seen by a dentist. The patient preference for seeking physicians initially, rather than dentists, concurs with published results in the literature.^4^^,^^9^

The time elapsed between perception of the first symptoms and arrival at the reference hospital in Alagoas showed that this time period was 1-3 months for most patients, followed by over 9 months in a smaller proportion. There was a proportional balance among these time periods, similar to reports in the literature;^9^^,^^27^^,^^30^^,^^31^ another study,^32^ however, found that the mean delay of patients was 273.19 days.

This situation reflects lack of information among patients; as lesions are initially asymptomatic,^33^ they only seek healthcare when tumors are at advanced stages, which incurs in drastically decreased survival.^28^

The referral time for professionals was mostly 2-6 months, which may be considered high, although coincident with published reports in the literature.^9^^,^^30^ Access to healthcare, the medical visit scheduling system, the transportation system, cultural and financial issues, and recognition of lesions by first-visit healthcare professionals are the main reasons for delayed referrals.^33^, ^34^, ^35^, ^36^, ^37^, ^38^, ^39^

Physicians or dentists referred mostly patients with advanced disease to a reference center; the referral rate of advanced cases was 56.8% for the former and 20.3% for the latter. These results suggest that lack of professional knowledge and preparedness about suspect mouth lesions increase the time before referral for these patients.

The role of the first healthcare professional to see these cases is extremely important, given the difficulty of diagnosing incipient malignancies;^40^ these professionals are responsible for referring such patients to specialized healthcare facilities.

Of 74 patients in the sample, 73.4% were referred to other professionals. More numerous visits before the diagnosis suggest a longer duration of complaints. An excessive number of healthcare professionals that were visited indicates that the patient will have seen many physicians and healthcare units and undergone many laboratory tests before a final diagnosis was made.

There are knowledge gaps about mouth cancer among healthcare professionals working in public health units, family health physicians and private clinics; there is, however, willingness to supplant these shortcomings by continued medical courses.^19^^,^^33^

Further points of debate in the literature are prevention and education strategies and professional training. The main concern focuses on undergraduate medical and dentistry courses.^3^

Lack of knowledge about mouth cancer among patients and healthcare professionals, fear of the diagnosis, and difficulties in accessing the healthcare system are important causes of delayed diagnoses. Healthcare professionals at times suggest that patients are responsible for their diseases. Such professionals are essential for diagnosing mouth cancer, and should be aware of this responsibility, rather than blaming patients for delays in the diagnosis. Successful therapy requires knowledge, positive attitudes, personal values, relationship abilities, psychological command, and self-confidence.^41^

Other factors were investigated in this study, such as the use of prostheses and difficulty in accessing healthcare services; no associations, however, were encountered, which became one of the shortcomings of this study.

The findings above suggest that continued education programs are needed for the population and for healthcare professionals to help identify the early signs of this disease, given its importance in defining the prognosis.

## CONCLUSION

Based on the results, we concluded that: There are no major differences between our results and those of other studies; the social and economical status of patients was low, and they had little or no information about the symptoms of mouth cancer and its prevention; male, rural worker, black patients aged over 60 years that smoked and drank alcohol were the most affected group; the most frequent anatomical site was the tongue, followed by the soft palate; there were many patients with advanced lesions, probably because initial tumors are asymptomatic, and because of social hurdles and lack of information; because stage III and IV tumors predominated, preventive, early diagnostic and disease control measures need to be optimized; diagnostic delays occurred mostly because of lack of information in patients, rather than healthcare professionals, although a significant number of these professionals were unprepared or lacked knowledge about suspected mouth lesions, which delayed patient referral; the number of patients that sought general practitioners was higher than those that sought specialists or dentists, which delayed referral since those healthcare professionals are more likely not to recognize the signs and symptoms of mouth cancer.
